# miRNA-1284, a regulator of HMGB1, inhibits cell proliferation and migration in osteosarcoma

**DOI:** 10.1042/BSR20171675

**Published:** 2018-07-13

**Authors:** Shuai Lv, Meng Guan

**Affiliations:** 1 Department of Gastroenterology, The First Affiliated Hospital of Zhengzhou University, Zhengzhou 450052, Henan province, China; 2 Department of Ophthalmology, The First Affiliated Hospital of Kunming Medical University, Kunming 650032, Yunnan Province, China

**Keywords:** cell proliferation, cell migration, HMGB1, miR-1284, Osteosarcoma

## Abstract

Previous literatures have reported the role of human micro RNA-1284 (hsa-miR-1284, in short miR-1284) in diverse cancers. However, its biological function in osteosarcoma pathogenesis remains unknown. In the present study, we investigated the potential role of miR-1284 in osteosarcoma. Expression of miR-1284 and high mobility group box 1 (HMGB1) were examined in 80 tissues obtained from 40 patients. MiR-1284 level was measured in five osteosarcoma cell lines. Relative luciferase activity and HMGB1 expression were examined in MG-63 and U2OS cells transfected with wild-type or mutant 3′-UTR of HMGB1 in the presence of miR-1284 mimics or miR-NC. Cell viability, colony formation, and cell migration were measured in MG-63, U2OS and hFOB 1.19 cells, which were transfected with miR-1284 mimics or miR-NC. In the rescue experiments, recombinant HMGB1 plasmid was transfected into MG-63 and U2OS cells, and cell viability and migration were determined again. Our results indicated that relative level of miR-1284 was lower in tumor tissues compared with its adjacent tissues and it was found suppressed at lower levels in MG-63 and U2OS cell lines. Expression of HMGB1 is significantly elevated in tumor tissues and negatively correlated with miR-1284 expression. MiR-1284 exerted its function by directly binding to 3′-UTR of HMGB1 and regulates expression of HMGB1. The overexpression of miR-1284 inhibited the cell proliferation and migration, and altered the protein expression of epithelial–mesenchymal transition (EMT)-associated genes (E-cadherin, N-cadherin, Vimentin, and Snail), which was reversed by HMGB1 overexpression. In conclusion, miR-1284 can function as a new regulator to inhibit osteosarcoma cell proliferation and migration by targeting HMGB1.

## Introduction

Osteosarcoma, the most common malignant bone tumor, has a peak incidence mostly in the second decade of life. Thanks to multiagent chemotherapy together with surgical techniques, the 5-year survival for patients with localized or primary osteosarcoma remains 60–70% [1]. But, the 5-year survival rate for metastatic osteosarcoma patients is only 20%, and the therapeutic strategies currently used for metastatic and recurrent osteosarcoma have limited efficacy [2]. Better understanding of the pathogenesis and progression of osteosarcoma at molecular level may contribute to the development of molecular targeted therapy.

MicroRNAs are small, noncoding, and highly conserved RNAs which play crucial roles in tumorigenesis and tumor growth [3,4]. Numerous studies have demonstrated the role of miRNAs in pathogenesis and disease progression in osteosarcoma. Regulation of hsa-miR-132-3p [5], hsa-miR-195-5p [6], and hsa-miR-17-5p [7] was reported to be involved in osteosarcoma cell proliferation, migration, and invasion. Recently, miR-1284 was demonstrated to play an important role in gastric cancer [8], ovarian cancer [9], and lung cancer [10]. To best of our knowledge, the roles of miR-1284 in osteosarcoma have not been previously investigated.

High mobility group box 1 (HMGB1), a ubiquitous nuclear protein, regulates various DNA-related activities such as repair, translation, replication, and recombination [11]. Impaired HMGB1 is associated with the cancer suggesting the potential as a target for cancer therapy [12]. Blocking HMGB1 release and activity reduces tumor incidence, invasion, and metastasis [13]. A few studies have demonstrated that HMGB1 is regulated by miRNAs in osteosarcoma [14–16]. In the present study, we aim to investigate potential role of miR-1284 in osteosarcoma cell growth and migration through regulation of HMGB1.

## Materials and methods

### Ethics statement

The study was approved by the Ethics Board of First Affiliated Hospital of Kunming Medical University and in compliance with the Declaration of Helsinki. All the patients gave their written informed consent.

### Human tissue samples and cell lines

In the study, 40 paired fresh frozen tissues from 40 patients were included, which were histologically confirmed osteosarcomas. The bone tissues adjacent to tumor tissues served as controls. Human osteoblast cell line hFOB 1.19 and human osteosarcoma cell lines, MG-63, U2OS, SAOS-2, G292, and SOSP-9607, were obtained from the Cell Bank of the Chinese Academy of Sciences (Shanghai, China). Cell lines were maintained in DMEM (Dulbecco’s modified Eagle’s medium, Invitrogen) supplemented with 10–15% fetal bovine serum (FBS) plus 2 mM l-glutamine and a penicillin–streptomycin cocktail at 37°C and 5% CO_2_ according to ATCC recommendation.

### Quantitative RT-PCR (reverse transcriptase polymerase chain reaction)

Total RNA was extracted from tissues and cells using TRIzol (Invitrogen Corporation, Carlsbad, CA, U.S.A.). TaqMan^®^ microRNA reverse transcription kit (Applied Biosystems^®^), and 15 ng of the RNA was used for reverse transcription. Real-time quantification polymerase chain reaction (qPCR) analysis was carried out after cDNA was synthesized. Reaction conditions were as follows: 95°C for 10 min; followed by 40 cycles of 95°C for 15 s, 60°C for 15 s and 72°C for 45 s for the amplification. The threshold cycle (*C*_t_) is defined as the fractional cycle number at which the fluorescence passes the fixed threshold. The gene expression *C*_t_ values of miRNAs from each sample were calculated by normalization to the internal control U6 snRNA. All experiments were repeated in triplicate. The primers used for PCR are listed as follows (F= forward; R=reverse):

**miR-1284**: F, 5′-CGTCTATACAGACCCTGGCTTTTC-3′, R, 5′-CTCAACTGGTGTCGTGGA-3′;
**U6**: F, 5′-TTATGGGTCCTAGCCTGAC-3′, R, 5′-CACTATTGCGGGTCTGC-3′;
**HMGB1**: F, 5′-TGGTATTTTGGACTGCGGGG-3′, R, 5′-TGACATTTTGCCTCTCGGCT -3′;
**GAPDH**: F, 5′-TGAAGGTCGGAGTCAACGG-3′, R, 5′-TCCTGGAAGATGGTGATGGGA-3′.

### Cell line transfection

Human osteosarcoma cell lines MG-63 and U2OS were placed onto a 60-mm dish, and after the cells had grown and reached to about 70% confluence, miR-1284 mimics, negative controls (miR-NC), miR-1284 mimics (Gene Pharma, Shanghai), and human HMGB1 recombinant plasmid (pPB-His-GST, Applied Biological Materials Inc, PV364047) were transfected into cells using Lipofectamine 2000 (Invitrogen), according to the manufacturer’s instructions. After 48 h of transfection, the cells were collected for further analysis.

### Luciferase reporter assays

Wild-type (WT) HMGB1 3′-UTR and mutant (MUT) HMGB1 3′-UTR luciferase reporter vector (Gene Pharma, Shanghai) were cotransfected with either miR-1284 mimic or negative controls (miR-NC). Human osteosarcoma cell lines MG-63 and U2OS were seeded into 24-well plates prior to incubation for 24 h. The cells were transfected with 0.1 μg WT 3′-UTR + 0.3 μg miR-1284 mimics, 0.1 μg MUT 3′-UTR + 0.3 μg miR-1284 mimics, 0.1 μg WT 3′-UTR + 0.3 μg miR-NC, and 0.1 μg MUT 3′-UTR + 0.3 NC. After 48 h of incubation, firefly luciferase luminescence activity was assayed using the Dual Luciferase Reporter Assay system (Promega, Madison, WI, U.S.A.). Renilla luciferase luminescence was then detected after adding Stop & Glo^®^ reagent (Promega) to each well. Luminescence was calculated as follows: relative luciferase activity = firefly luciferase luminescence/Renilla luciferase luminescence. Each evaluation was processed in triplicate.

### Western blot analysis

To extract the proteins, MG-63 and U2OS cells were washed twice in cold PBS, and then lysed in RIPA lysis buffer (Beyotime Institute of Biotechnology Jiangsu, China) with protease inhibitor cocktail (Merk, Germany). Protein concentration was quantified by BCA kit (Beyotime Institute of Biotechnology Jiangsu, China) and 50 μg of proteins were separated by SDS-PAGE on 8% gels, and then transferred to a polyvinylidene fluoride (PVDF) membrane (Millipore, U.S.A.). After blocking with 5% skim milk in 0.5% TBS-Tween-20 (v/v) for 1 h at room temperature, the membranes were incubated with the appropriate primary antibodies overnight at 4°C, including anti-HMGB1 (#ab79823, 1:10000), anti-E-cadherin (#ab1416, 1:50), anti-N-cadherin (#ab18203, 1:500), anti-Vimentin (ab92547, 1:2000), anti-Snail (ab53519, 1:1500), and anti-GAPDH (ab9485, 1:2500) antibodies. Primary and secondary antibodies were purchased from Abcam (Abcam, Cambridge, U.K.). Membranes were then washed once and incubated with antimouse secondary antibodies for 2 h at room temperature. Protein bands were visualized by using WEST-ZOL (plus) Western Blot Detection system (Intro Biotechnology, Inc., Seongnam, Korea).

### Cell proliferation assay

Cells were seeded into 96-well plates and 10 μl of CCK-8 was added to each well, and the cells were incubated with miR-NC, miR-1284 mimics at 37°C for 24, 48, 72 and 96 h respectively. Absorbance was analyzed on a 96-well Opsys MR Microplate Reader (Thermo Labsystems, Beverly, MA, U.S.A.) at 450 nm. All experiments were tested in triplicate and repeated at least three times.

### Colony formation assay

MG-63 and U2OS cells plated in triplicate at 500 cells per well in six-well plates were treated with miR-NC or miR-1284 mimics for 48 h. The cells were then incubated for 14 days and fixed with 4% paraformaldehyde (Beyotime, Haimen, China) and stained with Crystal Violet (Sigma-Aldrich). The colony formations (≥50 cells) were counted using an Olympus microscope IX73 (Olympus, Tokoyo, Japan).

### Cell migration assay

The Transwell chamber was washed and then divided into upper and bottom chambers by the Transwell insert. MG-63 and U2OS cells were transferred into the upper chambers. The lower chamber was filled with 500 ml DMEM supplemented with 10% FBS. After 24 h at 37°C under 5% CO_2_, cell migration was determined by counting cells in the bottom of the membrane stained with Crystal Violet, and scored visually in five random fields using light microscopy (magnification, ×100). The relative number of cells was calculated from the optical density (OD) value using an OSE-260 spectrophotometer.

### Statistical analysis

Data are expressed as mean ± standard deviation of at least three independent experiments. Statistical significance was assessed using student’s *t*-test or NAOVA (analysis of variance) where applicable. *P*<0.05 was considered to indicate a statistically significant difference.

## Results

### MiR-1284 is down-regulated in osteosarcoma tissue samples and MG-63 and U2OS cells

**Figure 1:**
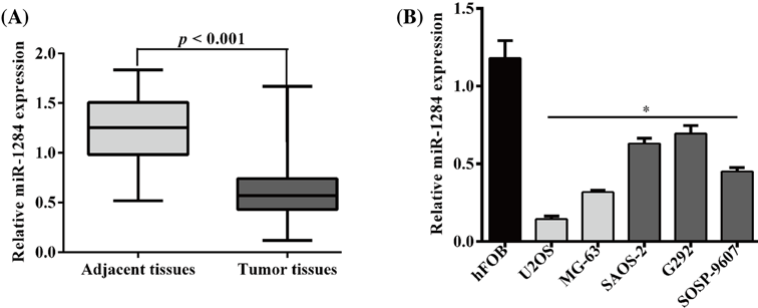
Expression level of miR-1284 in osteosarcoma tissue specimens and cell lines (**A**) Relative miR-1284 expression in tumor and tumor adjacent tissue samples. (**B**) Relative miR-1284 expression in six different cell lines, including hFOB 1.19, U2OS, MG-63, SAOS-2, G292, and SOSP-9607; *P<0.05, compared with hFOB 1.19.

Expression of miR-1284 in 40 osteosarcoma tissue samples and 40 matched nontumor normal tissue samples was detected by quantitative RT-PCR (*P*<0.001) (Figure 1A). Human osteoblastic cell line hFOB 1.19 was used as normal control. Relative miR-1284 expression in six different cell lines, including hFOB 1.19, U2OS, MG-63, SAOS-2, G292, and SOSP-9607 was shown in Figure 1B. Expression level of miR-1284 was lowest in U2OS and second lowest in MG-63 cell lines. Therefore, we selected these two cell lines for further *in vitro* examination.

### HMGB1 gene was down-regulated in tumor tissues and negatively correlated with miR-1284

**Figure 2:**
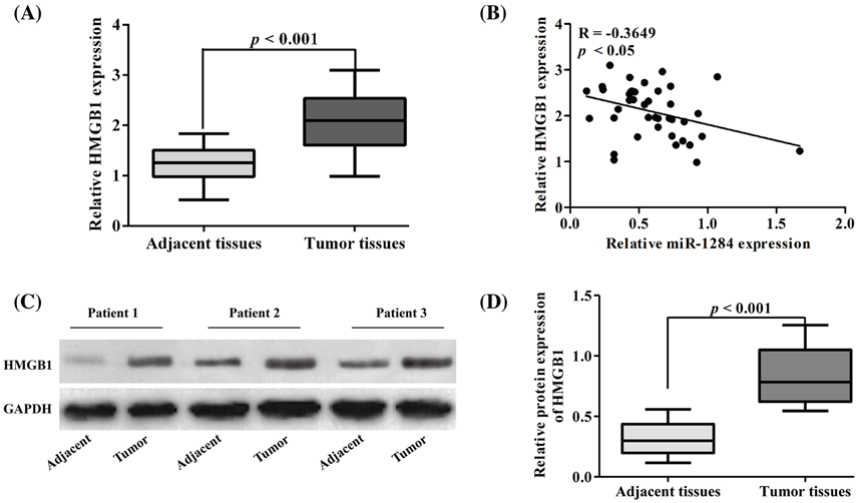
Relative expression level of HMGB1 in osteosarcoma tissue specimens and its correlation with miR-1284 (**A**) Relative mRNA expression of HMGB1 in tumor and paired-matched adjacent tissue samples. (**B**) Negative correlation between HMGB1 and miR-1284. (**C**) Protein expression of HMGB1 in three pairs of representative tumor and adjacent tissue samples measured with Western blotting. (**D**) Semiquatitative results of HMGB1 protein level based on densitometry of Western blots.

Our quantitative RT-PCR and Western blot analyses revealed that relative HMGB1 gene expressions at both transcriptional and translational levels were significantly higher in tumor tissues compared with its normal control tissues (*P*<0.001) (Figure 2A,C,D). In these tissue samples, relative HMGB1 mRNA expression was negatively correlated with miR-1284 expression level (*P*<0.05, *R* = −0.3649) (Figure 2B).

### HMGB1 is a target of miR-1284 in osteosarcoma cell lines

**Figure 3:**
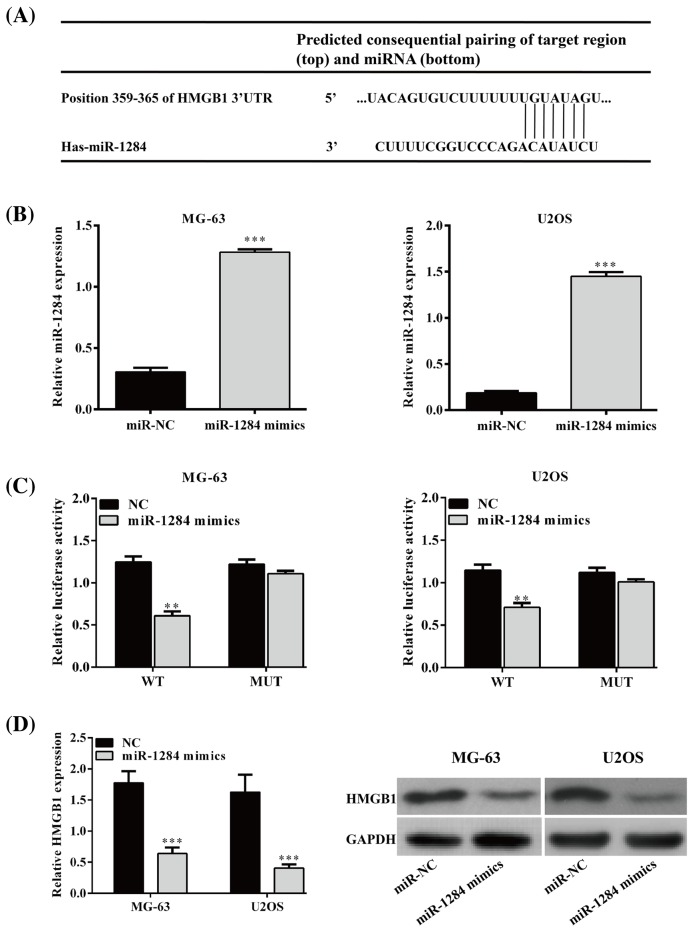
MiR-1284 directly targets HMGB1 by binding its 3′-UTR (**A**) The predicted miR-1284 binding site within HMGB1 3′-UTR and the mutant version. (**B**) Level of miR-1284 in the cells treated with miR-NC or miR-1284 mimics. (**C**) Luciferase reporter assay illustrating direct binding of miR-1284 to the WT, but not Mut sequences within the 3′-UTR of HMGB1. (**D**) mRNA and protein levels of HMGB1 in MG-63 and U2OS cells were determined by quantitative RT-PCR and Western blot analyses. **P<0.01; ***P<0.001.

Based on the miRNA database (TargetScan, http://www.targetscan.org/vert_72/; miRDB, http://www.mirdb.org/), HMGB1 is a predicted target of miR-1284 in humans (Figure 3A). We used miR-1284 mimics and negative control (miR-NC) to determine the effect of overexpressed miR-1284 on the expression of HMGB1 in human osteosarcoma cell lines, MG-63, and U2OS. MiR-1284 mimics increased the level of miR-1284 (Figure 3B) and significantly inhibited HMGB1 luciferase activities in the two types of cells (*P*<0.05), whereas miR-1284 mimics had no effect on the expression of the luciferase reporter containing HMGB1-3′-UTR with mutated miR-1284 binding sites (HMGB1-MUT) in both cell lines (Figure 3C). The mRNA level of HMGB1 significantly decreased following miR-1284 mimic treatment compared with its negative controls (*P*<0.001) (Figure 3D). Consistently, the protein levels of HMGB1 decreased following miR-1284 mimics treatment compared with miR-NC in MG-63 and U2OS cells (Figure 3D). These findings suggested that HMGB1 is the target of miR-1284.

### Overexpression of miR-1284 inhibits cell growth and cell migration in MG-63 and U2OS cell lines

**Figure 4:**
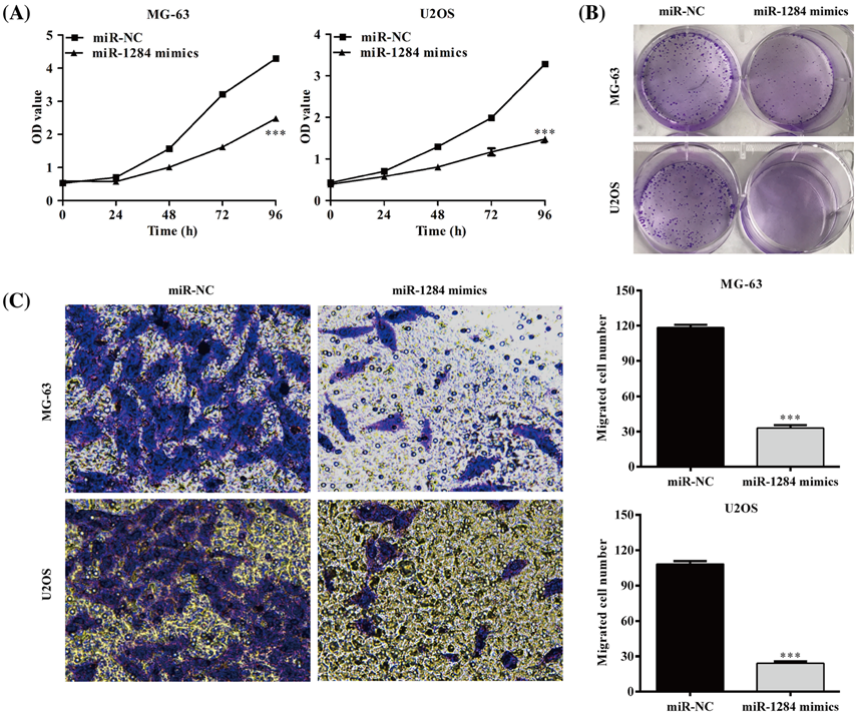
MiR-1284 overexpression inhibits osteosarcoma cell proliferation and migration (**A**) Cell viability in both MG-63 and U2OS cells transfected with miR-1284 mimics and negative control. (**B**) Colony formation activity in MG-63 and U2OS cells transfected with miR-1284 mimics and negative control. (**C**) Cell migration analysis in MG-63 and U2OS cells transfected with miR-1284 mimics and negative control. ***P<0.001, compared with miR-NC.

The proliferation rate of MG-63 and U2OS cells with or without miR-1284 mimics treatment was determined using CCK-8 assay. The cells transfected with miR-1284 mimics exhibited significant reduction in proliferation compared with the cells transfected with miR-NC (*P*<0.001), in both MG-63 and U2OS cell lines (Figure 4A). Colony formation assay confirmed the results of CCK-8 assay. Overexpression of miR-1284 inhibits colony formation ability of MG-63 and U2OS cells (Figure 4B). Transwell migration results indicated that the number of MG-63 or U2OS cells migrated into Transwell inserts on the lower surface of the inserts and into the bottom chamber was significantly higher in the miR-1284 overexpression group compared with negative control group (*P*<0.001) (Figure 4C).

### Inhibition of miR-1284 promotes the growth and migration of hFOB 1.19 cells

**Figure 5:**
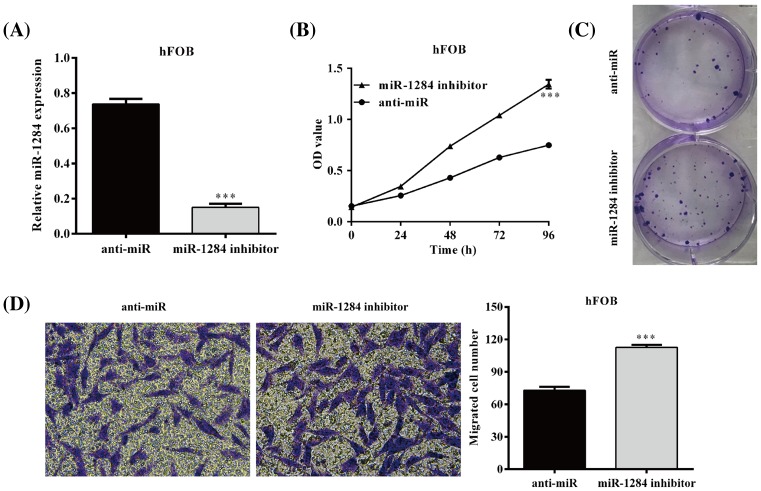
MiR-1284 inhibition promotes osteoblastic cell proliferation and migration (**A**) MiR-1284 level after its specific inhibitor was transfected in hFOB 1.19 cells. (**B**) Viability of hFOB 1.19 cells after transfection with miR-NC or miR-1284 inhibitor. (**C**) Colony formation assay performed after the cells transfection with miR-NC or miR-1284 inhibitor. (**D**) Transwell migration of the cells transfected with miR-NC or miR-1284 inhibitor. ***P<0.001, compared with miR-NC.

The specific inhibitor of miR-1284 effectively decreased miR-2184 in hFOB 1.19 cells and promoted their viability (Figure 5A,B). The results of colony formation showed remarkably increased colonies of hFOB 1.19 cells after the transfection with miR-1284 inhibitor (Figure 5C). Transwell migration assay proved that miR-1284 inhibition resulted in significantly increased number of migrated cells, compared with the cells transfected with miR-NC (*P*<0.001; Figure 5D).

### HMGB1 overexpression rescues the growth and migration of MG-63 and U2OS cells inhibited by miR-1284

**Figure 6:**
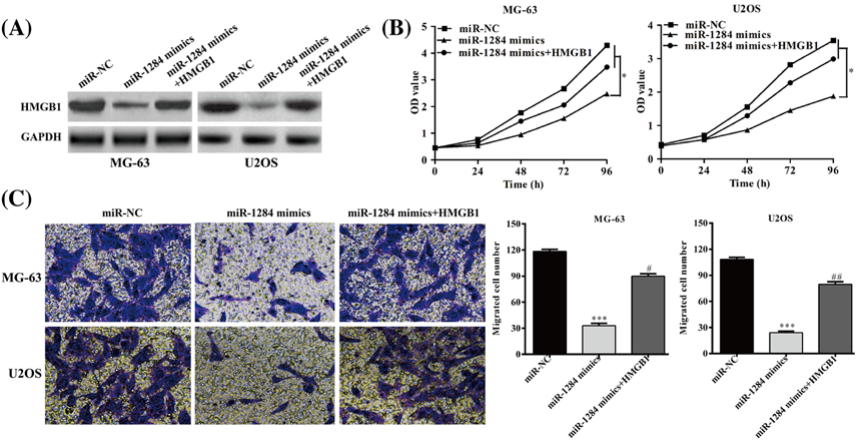
HMGB1 is involved in miR-1284-mediated inhibition of cellular growth and migration (**A**) HMGB1 protein expression in MG-63 and U2OS cells transfected with miRNA negative control, miR-1284 mimics, or miR-1284 mimics + HMGB1-carrying vector. (**B**) Cell viability in cells with different transfection. (**C**) Cell migration analysis in cells with different transfection. *P<0.05, ***P<0.001, vs. NC; ^#^*P*<0.05, ^##^*P*<0.01, vs. miR-1284 mimics.

To clarify that miR-1284 plays an important role in the inhibition of cellular growth and migration through the regulation of HMGB1, we performed rescue experiment by cotransfecting the cells with miR-1284 mimics and HMGB1. As shown in Figure 6A, HMGB1 was overexpressed in HMGB1 transfected cells. In addition, we found that HMGB1 restored the cell proliferation and migration abilities that reduced by miR-1284 mimics (Figure 6B,C). Taken together, our results suggest that HMGB1 may plays an important role in miR-1284 mimics-mediated reduction of osteosarcoma cell growth and metastasis.

### HMGB1 overexpression restores the expression of miR-1284/HMGB1 downstream EMT-associated proteins

**Figure 7:**
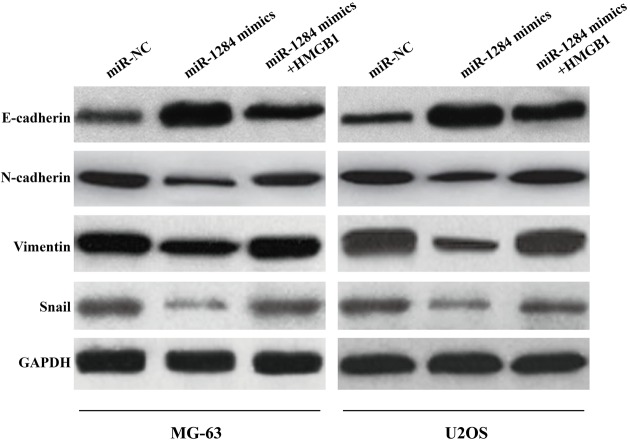
EMT-associated proteins in the downstream of miR-1284/HMGB1 was regulated by miR-1284 and can be restored by HMGB1 overexpression EMT-associated genes, including E-cadherin, N-cadherin, Vimentin and Snail, in MG-63 and U2OS cells were measured by western blotting.

As shown in Figure 7, the protein levels of E-Cadherin, N-Cadherin, Vimentin, and Snail in the cells treated with miR-NC, miR-1284 mimics, or miR-1284+HMGB1 were measured with Western blotting. The results showed the notably decreased level of N-cadherin, Vimentin, and Snail, but remarkably increased level of E-cadherin after the osteosarcoma cells (both MG63 and U2OS cell lines) were treated with miR-1284 mimics. However, the levels of these proteins were restored in the presence of the overexpressed HMGB1.

## Discussion

Altered expression of miRNAs is associated with gene regulation, cellular signaling, and metastasis in human carcinogenesis [17–19]. A miRNA can target multiple genes, and reversely, a gene can be targeted by multiple miRNAs. In human carcinogenesis, aberrant miRNA expression correlates with diverse human cancers. This indicates that miRNAs can function as tumor suppressors and/or oncogenes through altering the expression of important cancer-related genes [14,20]. Previously, it was reported that overexpression of HMGB1 is associated with tumorigenesis, invasion, and metastasis of osteosarcoma, and its expression can be regulated by multiple miRNAs in diverse cancers [14,16,21]. However, no report ever describes the regulatory role of miR-1284 in osteosarcoma or its correlation with HMGB1 in any carcinogenesis.

In the present study, we showed that miR-1284 was down-regulated in osteosarcoma tissues where HMGB1 is overexpressed, indicating a reverse correlation between miR-1284 and HMGB1. In addition, miR-1284 was directly binding to 3′-UTR region of HMGB1 and led to its degradation. Relative mRNA and protein expression of HMGB1 was significantly lower in presence of overexpressed miR-1284. miRNA-1284 inhibited the cell proliferation and migration in both MG-63 and U2OS cell lines and this inhibition was abolished with addition of HMGB1.

Several years ago, miR-1284 was first reported to be the most frequently expressed circulating miRNA, suggesting the potential ability to distinguish lung cancer cases from clinically relevant controls [22]. Subsequent study on miRNA profiling also reported that miR-1284 was down-regulated in paired lymph node metastases compared with primary gastric cancers [23]. These literatures have established the fundamental purpose for the following functional experiments. Increased cell viability and growth, and decreased apoptosis were observed in lung cancer A549 cells which were transfected with miR-1284 mimics [10]. Inhibition of proliferation and induction of apoptosis were caused by miR-1284’s inhibiting Bcl-2 expression in SGC-7901 gastric cancer cells [24]. MiR-1284 could inhibit cell viability via regulating the expression of p27 and induce apoptosis via regulating the PI3K/Akt pathway in OVCAR3 ovarian cancer cells [9]. The expression of miR-1284 was reduced in gastric cancer (GC) tissue specimens with metastasis and vincristine-resistant (VCR) GC SGC7901 cells, and overexpression of miR-1284 reversed the chemoresistance of SGC7901/VCR cells by targeting EIF4A1 [8].

Sustained levels of HMGB1 aid tumorigenesis and are crucial for metastasis [12]. Dysregulation of HMGB1 is linked to several types of cancer including osteosarcoma. It was reported that hsa-miR-22-3p inhibits osteosarcoma cell proliferation and migration through targeting HMGB1 [25]. Long noncoding RNA (LncRNA) metastasis-associated lung adenocarcinoma transcript 1 (MALAT1), an lncRNA with notorious roles in multiple aggressive tumors, was reported to promote osteosarcoma development by up-regulation of HMGB1 via hsa-miR-142-3p and hsa-miR-129-5p [26]. In the downstream of the miR-1284/HMGB1 signal axis, expression of the genes associated with epithelial–mesenchymal transition (EMT), which enables tumor cells to migrate and invade [27,28], was measured with Western blotting. E-cadherin is the most well-documented member of the cadherin family that helps cell adhesion. N-cadherin is a transmembrane protein expressed in various tissues and mediates cell–cell adhesion, playing a role in neurons and cancer metastasis. Studies have shown that the switch from E-cadherin to N-cadherin contributes greatly to EMT phenotype and the progress of various cancers [29–31]. Vimentin is a fragment of intermediate filament, and it is also an EMT marker mediating migration of tumor cells [32]. Snail is capable of inducing EMT by directly repressing the transcription of E-cadherin [33]. Our results showed that overexpressed miR-1284 in both MG-63 and U2OS cells significantly inhibited the HMGB1 mRNA and protein expression and further resulted in a reduced cell proliferation and migration of osteosarcoma cells, which might be caused by the increase in E-cadherin protein level and decrease in N-cadherin, Vimentin, and Snail protein levels.

In conclusion, our results exhibited that miR-1284 is able to target HMGB1, inhibiting the cell proliferation and migration, and suppress EMT of the osteosarcoma. These findings suggest that the lower level of miR-1284 may have a crucial role in osteosarcoma carcinogenesis and miR-1284/HMGB1 axis may be a potential therapeutic target in the treatment of osteosarcoma.

## Abbreviations

EMT: epithelial–mesenchymal transition
FBS: fetal bovine serum
HMGB1: high mobility group box 1
lncRNA: long noncoding RNA
MALAT1: metastasis-associated lung adenocarcinoma transcript 1
miRNA: micro RNA
MUT: mutant
NC: negative control
OD: optical density
qPCR: quantification polymerase chain reaction
VCR: vincristine-resistant
WT: wild-type
